# Supplementary material for: Exploration of street space architectural color measurement based on street view big data and deep learning—A case study of Jiefang North Road Street in Tianjin
Source: PLoS One. 2023 Nov 30;18(11):e0289305. doi: 10.1371/journal.pone.0289305 (PMC10688869; doi:10.1371/journal.pone.0289305)
Supplement: Attachment — Submitted filename: Review Report of Manuscript PONE.pdf [file pone.0289305.s001.pdf]

Review Report of Manuscript PONE-D-23-07210

"Exploration of street space architectural color measurement based on street view big data and deep learning-- A case study of Jiefang North Road Street in Tianjin"

General Comments:

The Research objective of providing a machine learning based tool for street colour measuring is interesting for many future applications, especially in retail and entertainment architecture applications.

The research methodology, results, and analysis are sufficient for the proposed case.

Recommendations

General English language and Style is recommended.

Decision

Accept
