# Supplementary material for: Exploration of street space architectural color measurement based on street view big data and deep learning—A case study of Jiefang North Road Street in Tianjin
Source: PLoS One. 2023 Nov 30;18(11):e0289305. doi: 10.1371/journal.pone.0289305 (PMC10688869; doi:10.1371/journal.pone.0289305)
Supplement: Attachment — Submitted filename: 0712Response to Reviewers.docx [file pone.0289305.s003.docx]

**Cover Letter**

Dear Editor,

We appreciate you and the reviewers for your precious time in reviewing our manuscript" Exploration of street space architectural color measurement based on street view big data and deep learning-- A case study of Jiefang North Road Street in Tianjin” (PONE-D-23-07210). It was your valuable and insightful comments that led to possible improvements in the current version.

The authors have carefully considered the comments and tried our best to address every one of them. We hope the manuscript after careful revisions meet your high standards. The authors welcome further constructive comments if any.

Below we provide the point-by-point responses. All modifications in the manuscript have been marked page and line numbers in response.

In this study, funder Shibao Yu participated in the study design and manuscript preparation. The funding is "Young Scholars Science Foundation of Lanzhou Jiaotong University (2020033) ".

In this study, the sponsor Hongxu Peng participated in the study design and data analysis. The funding is Fujian Provincial Social Science Planning Project "Study on the Influence and Significance of Zhu Zi Studies on Korean Confucian Habitat Culture"(Grant No.FJ2021C035).

We have redrawn Figure 2, Figure 8, and Figure 9, removing the all of base maps. As such, they are not subject to the restrictions of the Creative Commons Attribution License (CC by 4.0).

We deleted all images from Baidu Map in the article to avoid commercial licensing issues. Images 3, 4, 5, and 6 were taken with a DSLR camera at our study area. The author of this article owns all copyright of the source file of this image.

All modifications in the manuscript have been marked page and line numbers in response. We checked the references one by one and removed and corrected those that did not meet the criteria.

I have removed the fund section from the manuscript. The fund statement remains unchanged.

If our revisions still cannot meet the requirements, please do not hesitate to contact us. We are more than willing to continue making modifications until it reaches the level and standards required for journal publication.

Best regards,

Mr. Lei Wang

Email: wanglei2021@tju.edu.cn

School of Architecture

Tianjin University

**Response to Journal**

**[Journal Requirements]** Please review your reference list to ensure that it is complete and correct. If you have cited papers that have been retracted, please include the rationale for doing so in the manuscript text or remove these references and replace them with relevant current references. Any changes to the reference list should be mentioned in the rebuttal letter that accompanies your revised manuscript. If you need to cite a retracted article, indicate the article’s retracted status in the References list and also include a citation and full reference for the retraction notice.

**Response:** Thanks for your kind reminders. We checked the references one by one and removed and corrected those that did not meet the criteria.

5. Zhang J, Fukuda T, Yabuki N. A Large-Scale Measurement and Quantitative Analysis Method of Façade Color in the Urban Street Using Deep Learning. In: Yuan PF, Yao J, Yan C, Wang X, Leach N, editors. Proceedings of the 2020 DigitalFUTURES. Singapore: Springer; 2021. pp. 93–102. doi:10.1007/978-981-33-4400-6_9

→ 5. Zhang J, Fukuda T, Yabuki N. A Large-Scale Measurement and Quantitative Analysis Method of Façade Color in the Urban Street Using Deep Learning. Proceedings of the 2020 DigitalFUTURES. Singapore: Springer Singapore; 2021. pp. 93–102. doi:10.1007/978-981-33-4400-6_9 **[Pg34, Line 555-558]**

Removed non-compliant references 11(11.	Cavallo M. 3D City Reconstruction From Google Street View. : 6.)

**Response to Reviewer 3**

**[General Comment]** From the prior comments, it appears that the authors improved and corrected the paper as well as included some justifiable arguments and supplements. However, there are still a few minor issues that the author should be to improvement. Such as the insertion of authors? Is it permissible to have 8 corresponding authors and 2 co-authors? Section numbers are missing, etc.

**Response:** Thank you for your valuable comments on our paper and for taking the time to read and review it carefully. We have thought deeply about the questions you raised and will now answer and explain each of them.

You mentioned the question of whether our article is allowed to have 8 corresponding authors and 2 co-authors. These authors are the ones who made important contributions during the late revision process of the paper. Their efforts were crucial to our research, for which we are deeply grateful. To reflect their contributions, we have chosen to add them as corresponding authors and co-authors. We understand that this can be problematic and have made every effort to ensure that each author's contribution is clearly articulated in the paper. Regarding the issue of chapter numbers, it is our understanding that the standard format of this journal does not require chapter numbers. We appreciate your suggestion, which is very valuable to us. We will do our best to improve and optimize our work to ensure its final quality.

**Response to Reviewer 4**

**[General Comment]** After studying the contribution, it can be concluded that the authors approached it responsibly when editing it. They corrected everything he was reminded of. They edited the text to make it more understandable and clearer. In this form, I recommend accepting the contribution.

**Response:** Thank you very much for your recognition and positive comments on my paper. I am very pleased to receive your approval, which has had a very positive impact and motivation on my research work in this area. Your expertise and careful review have made it possible to present this paper in a better way, for which I am sincerely grateful. I will continue to work hard, maintain a humble and cautious attitude in my research, and strive to expand the body of knowledge in this field to contribute more to the academic community.
